# Primary Chondrosarcoma in L-shaped Crossed Fused Renal Ectopia Coexisting with Papillary Urothelial Carcinoma in Urinary Bladder – An Enigmatic Entity with Poor Prognosis

**DOI:** 10.15586/jkcvhl.v9i1.162

**Published:** 2022-01-01

**Authors:** Mayank Kumar, Aasma Nalwa, Taruna Yadav, Poonam Elhence, Himanshu Pandey, Meenakshi Rao

**Affiliations:** 1 Department of Pathology, Autonomous State Medical College, Ayodhya, India;; 2 Department of Pathology, All India Institute of Medical Sciences, Jodhpur, India;; 3 Department of Radiology, All India Institute of Medical Sciences, Jodhpur, India;; 4 Department of Urology, All India Institute of Medical Sciences, Jodhpur, India

**Keywords:** chondrosarcoma, kidney, renal ectopia, urinary bladder, urothelial carcinoma

## Abstract

Primary renal chondrosarcomas are rare tumors that are high-grade in nature and, unfortunately, have poorly understood pathogenesis and extremely low prognosis. The coexistence of a discrete malignancy in the urinary bladder is even rarer, with the occurrence of distinct papillary urothelial carcinoma in the urinary bladder in this case. The clinical presentation is nonspecific, and the primary radiological investigations have a limited scope in providing specific diagnosis of this entity. The final diagnosis is possible on thorough histopathological examination of the resected specimen, which requires extensive sampling and meticulous reporting. As of now, the only way to achieve a better prognosis is by early diagnosis. It is necessary to keep the possibility of occurrence of sarcomas at rare sites in the differential diagnoses. The cytogenetic and molecular abnormalities associated with this entity need to be elucidated to achieve a more satisfactory outcome concerning the overall management of the patient.

## Introduction

Primary renal sarcomas are uncommon tumors, accounting for only 1%– 5% of all renal malignancies (1). Of these, primary renal chondrosarcomas are extremely rare, with only a few cases reported in the literature.

This high-grade malignancy has unique biphasic histology with poorly understood pathogenesis, disease course, and even poorer prognosis (2). Herein we describe the first case report documenting the occurrence of primary renal chondrosarcoma in L-shaped crossed fused renal ectopia that involves both moieties, along with a distinct papillary urothelial carcinoma in the urinary bladder.

## Case Report

A 50-year-old male presented with hematuria, increased frequency, and burning sensation during micturition along with left flank pain for 2 months. There was a history of generalized weakness, weight loss, and loss of appetite during this period. On examination, the patient was poorly nourished with the presence of a palpable left-sided abdominal mass.

Ultrasonography (USG) of the abdomen showed the presence of right ectopic kidney and left-sided hydronephrosis. A heterogeneous mass was noted in the left kidney. Another polypoidal mass was also seen in the lumen of the urinary bladder, attached to its posterolateral wall.

Contrast-enhanced computerized tomography (CECT) of the abdomen helped in the renal anatomy and characterization of the mass, which revealed L-shaped crossed fused renal ectopia. The right kidney was not present in the right renal fossa and was in the midline, anterior to the aortic bifurcation at the L4-L5 level. It was malrotated and fused with the lower pole of the left kidney. The left kidney was enlarged, with a large soft heterogeneous tissue density mass involving the interpolar and lower pole regions that exhibited heterogeneous enhancement with central non-enhancing areas. Few calcified foci were seen in the mass, along with moderate hydronephrosis. In the delayed phase (15 minutes), no contrast excretion from the left kidney was recorded. The interpolar region of the right kidney was contiguously infiltrated by the left lower pole renal mass. Small tumor thrombi were present in the segmental right renal veins draining the interpolar region. Aortocaval, para-aortic and left renal hilar lymphadenopathy were also noted.

Along with these findings, a well-defined polypoidal mass was seen in the left posterolateral wall of the urinary bladder, infiltrating the left vesicoureteral junction. The middle and distal parts of the left ureter were contiguously involved by this urinary bladder mass. A peripheral rim of calcification was present.

Because of the involvement of multifocal enhancing masses of the moieties of crossed fused renal ectopia, urinary bladder, and left ureter, the radiological differential diagnoses offered were multifocal transitional cell carcinoma, renal cell carcinoma (RCC)- mucinous adenocarcinoma variant with multifocal spread, and renal sarcoma.

No distant lesion was found on metastatic work-up.

**Figure 1:**
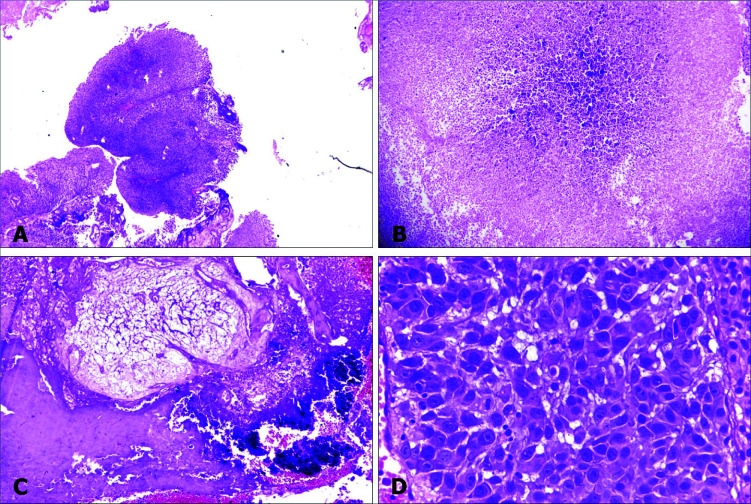
Tumor in urinary bladder. Photomicrographs of tumour in urinary bladder, showing noninvasive papillary urothelial carcinoma (A, 4x), area of necrosis (B, 10x), focus of calcification (C, 10x), and tumour (D, 40x)

Initially, transurethral resection of the bladder lesion was done. Microscopic examination showed features of noninvasive papillary urothelial carcinoma, predominantly low-grade with high-grade focal areas, along with extensive dystrophic calcification and necrosis, and focal osseous metaplasia (Figure 1).

**Figure 2:**
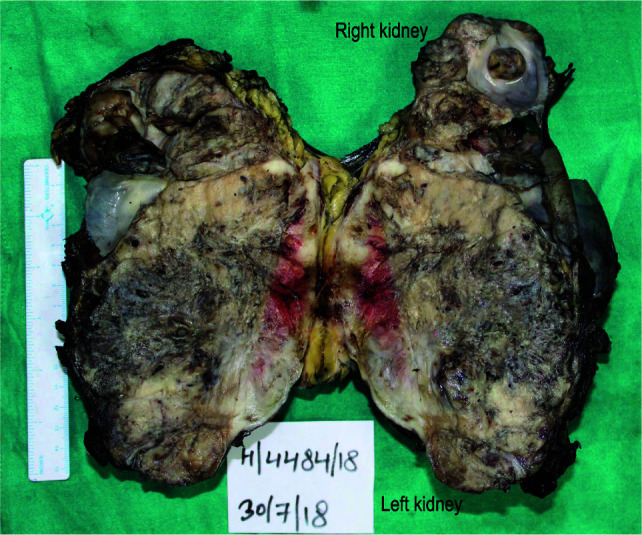
Gross image of the resected specimen. The photograph shows cut sections of left and right kidney, with extensive tumor.

The patient was then taken up for surgery for resection of the renal mass, and a left nephrectomy with partial right nephrectomy was also performed. The specimen was submitted for histopathological examination. The results showed that the capsule was intact. Cut section of the left kidney showed a tumor measuring 14x11x10 cm, replacing the entire normal structure. The renal pelvis was not identified, and part of the right kidney consisted of cystic and solid areas, with tumor measuring 3x2.5x2 cm (Figure 2).

**Figure 3:**
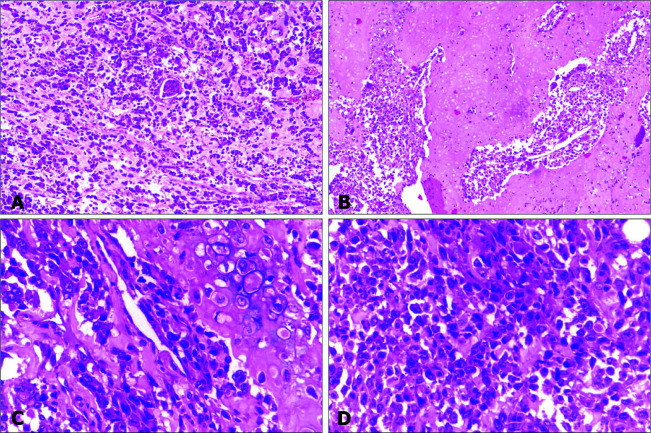
Tumor in kidney. Photomicrographs of tumour in kidney, showing tumour (A, 10x), areas of cartilaginous differentiation (B and C, 10x and 40x), and high mitotic activity (D, 40x).

Multiple sections examined from both the kidneys showed a tumor composed of large areas of cartilaginous differentiation along with tumor cells arranged in diffuse sheets and fascicles. Marked pleomorphism and mitotic activity were noted, 18-19/10hpf. There was an abrupt transition to well-differentiated nodules of hyaline cartilage. Intervening stroma showed consistent focal areas of myxoid change with chronic inflammatory cell infiltrate. The focal osteoid formation was present, with numerous giant cells and apoptotic debris. Areas of chicken-wire calcification and hemorrhage were also identified, along with large necrotic sections (Figure 3).

**Figure 4:**
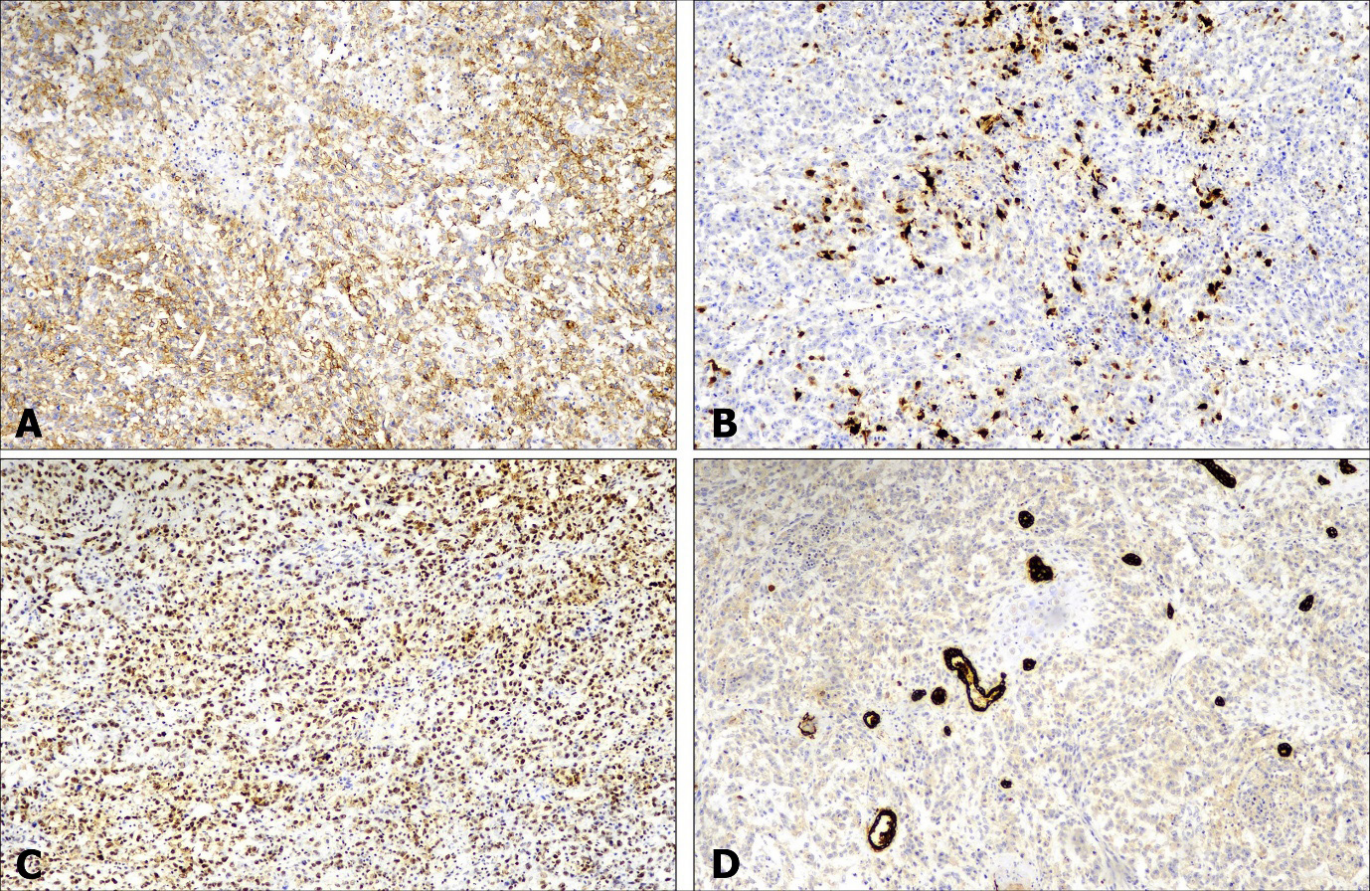
Immunohistochemical findings. Photomicrographs showing immunopositivity of tumor cells for CD99 (A) and S100 protein (B), retention of INI1 expression (C), and immunonegativity for pan cytokeratin (D).

**Figure 5:**
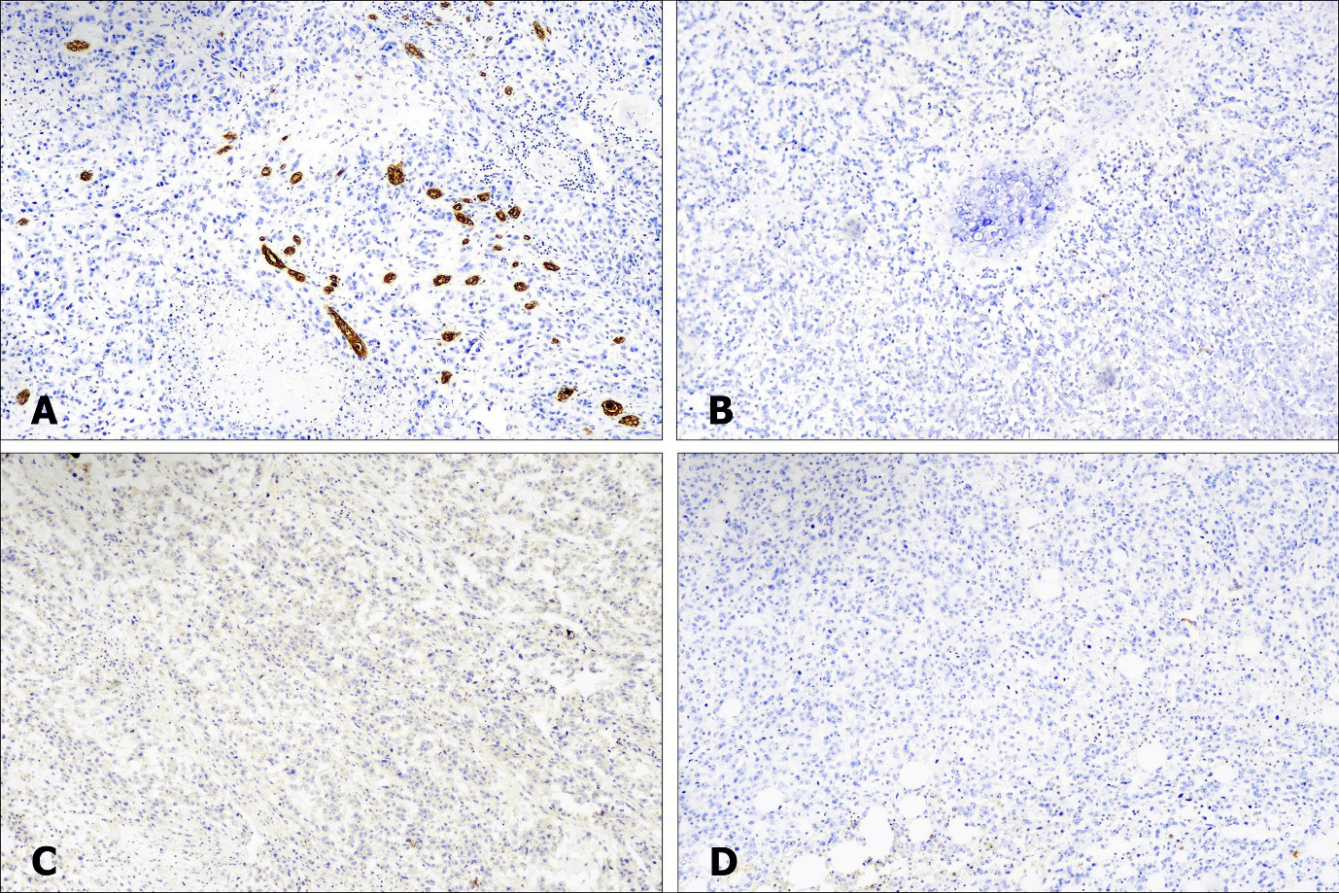
Immunohistochemical findings. Photomicrographs showing immunonegativity of tumor cells for CK7 (A), p63 (B), myogenin (C), and HMB45 (D).

The tumor cells were immunopositive for CD99 with strong S100 protein expression in the areas of cartilaginous differentiation, immunonegative for pan-cytokeratin, CK7, CK20, p63, desmin, and myogenin, and with retention of INI1 expression (Figures 4 and 5). These morphological and immunohistochemical features suggested the presence of primary chondrosarcoma in crossed fused renal ectopia involving both moieties.

The patient was lost to follow-up after discharge from the hospital after an uneventful postsurgical period of 2 weeks.

## Discussion

Mesenchymal chondrosarcomas, first reported in 1959, are rare neoplasms and constitute about 1% of all chondrosarcomas seen commonly in the skeleton, with one-third of the cases arising in soft tissues and other organs. The extraskeletal mesenchymal chondrosarcoma sites include the head and neck, lower extremities, trunk, and retroperitoneum (3,4). Primary renal chondrosarcoma is very rare, with only a handful of cases reported since the entity was first described in 1984 (5).

Although rare, the occurrence of renal chondrosarcoma as a primary lesion is not unexpected, as the kidney and cartilage have the same mesodermal origin (4). However, the exact histogenesis is uncertain, and because of rarity of the entity, there have been very few studies regarding pathogenesis.

The most widely accepted hypothesis is that tumor development occurs in two stages, with secondary malignant transformation in tissue scattered during embryogenesis. Recent theories suggest that this entity occurs because of the pathological mesenchymal differentiation in stem cells (1). Few studies have evaluated the role of mesenchymal-epithelial transition in the pathogenesis (6).

The tumor shows a characteristic growth, consisting of cellular undifferentiated tumor cell components and nests of well-differentiated cartilage. The transition from the undifferentiated component to the cartilage nests is usually abrupt. The differential diagnoses on light microscopy can include Ewing sarcoma, small cell osteosarcoma, dedifferentiated chondrosarcoma, and hemangiopericytoma (3). The entity may be confused with Wilm’s tumor as the undifferentiated tumor cells and cartilage can be mistaken as blastemal and mesenchymal components (7). However, the characteristic appearance and the absence of osteoid formation and immunohistochemical studies help in the diagnosis.

Extensive sampling and thorough histological examination are necessary to rule out the existence of RCC showing extensive mesenchymal differentiation with composite morphology. Sarcomatoid change inclusive of osteosarcomatous and chondrosarcomatous differentiation is not uncommon in RCCs and has been widely reported (8). In our case, even after examining multiple sections from various locations, there was no evidence of RCC.

Crossed fused renal ectopia is a rare congenital anomaly wherein the kidneys are fused and located on the same side of the midline. It is subclassified into various types based on the configuration of fusion abnormality. In this case, the presence of this anomaly and involvement of both moieties increased the surgical complexity. However, preoperative evaluation of the renal and vascular anatomy using imaging played a vital role in the overall management of the patient.

To the best of our knowledge, the occurrence of coexistent renal chondrosarcoma in crossed fused renal ectopia involving both moieties, with papillary urothelial carcinoma in the urinary bladder has not been reported earlier. A previous case report described these entities occurring as separate lesions in the same kidney (9). This association may be coincidental or can be a response to a common causative factor.

Imaging studies play an integral role in the evaluation of an abdominal mass. However, to date, no specific radiological features aid in the diagnosis of renal chondrosarcoma. The most common radiological finding is the occurrence of calcification within the tumor (1). Also, the diagnosis of primary renal chondrosarcoma necessitates exclusion of metastasis to the kidney from a skeletal chondrosarcoma by imaging.

Most of the cases of primary renal chondrosarcoma reported in literature had metastasis at initial presentation. The sites for metastasis include the liver, lung, ureter, thyroid gland, and femora (2,10). Simultaneous occurrence of mesenchymal chondrosarcoma in the kidney and spleen has also been reported (11).

There are very few studies regarding the cytogenetic and molecular abnormalities in renal chondrosarcomas. FLI-1 expression seen in Ewing sarcoma differentiates it from mesenchymal chondrosarcoma and small cell osteosarcoma (12). HEY1-NCOA2 fusion is considered to be diagnostic for mesenchymal chondrosarcomas (13). In bones, chondrosarcomas exhibit p53 mutations only in a minority of cases. When present, these occur in higher grade tumors (14).

Increased p53 expression is associated with an overall poorer prognosis in RCCs (15). However, there is a shortage of studies regarding the same concerning primary renal chondrosarcomas. Also, the association with hereditary syndromes has not been established, probably because of the rarity of the condition.

In the absence of a definite treatment protocol, radical surgical resection with adequate margins and adjuvant chemotherapy with multiple agents are used for management (16).

A possible chemotherapy regimen for adjuvant therapy is the combination of doxorubicin, vincristine, and cyclophosphamide, along with administration of etoposide and ifosfamide. The malignant mesenchymal cells show increased expression of platelet-derived growth factor receptor α (PDGFR-α). So agents that inhibit PDGFR-α function, such as dasatinib, sorafenib, and imatinib, may play a role in the therapy (17).

## Conclusion

The disease course of primary renal chondrosarcoma is poorly understood because of its rarity and poor survival rates. Elucidation of cytogenetic and molecular abnormalities will help in designing a definite treatment protocol. As of now, a better prognosis may be achieved by early diagnosis and prompt initiation of appropriate management. It is necessary to keep in mind the possible occurrence of sarcomas at rare sites because in such unusual cases, the period available for altering the disease outcome is limited.

## Consent

Written informed consent was obtained as per institutional guidelines.
